# Experimental Study of the Pyrolysis of NH_3_ under Flow Reactor Conditions

**DOI:** 10.1021/acs.energyfuels.0c03387

**Published:** 2021-01-20

**Authors:** Mario Benés, Guillermo Pozo, María Abián, Ángela Millera, Rafael Bilbao, María U. Alzueta

**Affiliations:** Aragón Institute of Engineering Research (I3A), Department of Chemical and Environmental Engineering, University of Zaragoza, 50018 Zaragoza, Spain

## Abstract

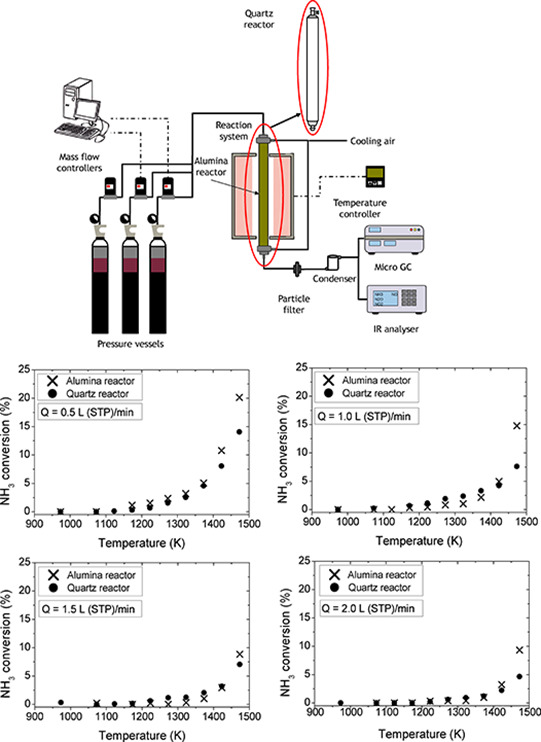

The possibility of using ammonia (NH_3_), as a fuel and as an energy carrier with low pollutant emissions, can contribute to the transition to a low-carbon economy. To use ammonia as fuel, knowledge about the NH_3_ conversion is desired. In particular, the conversion of ammonia under pyrolysis conditions could be determinant in the description of its combustion mechanism. In this work, pyrolysis experiments of ammonia have been performed in both a quartz tubular flow reactor (900–1500 K) and a non-porous alumina tubular flow reactor (900–1800 K) using Ar or N_2_ as bath gas. An experimental study of the influence of the reactor material (quartz or alumina), the bulk gas (N_2_ or Ar), the ammonia inlet concentration (1000 and 10 000 ppm), and the gas residence time [2060/*T* (K)–8239/*T* (K) s] on the pyrolysis process has been performed. After the reaction, the resulting compounds (NH_3_, H_2_, and N_2_) are analyzed in a gas chromatograph/thermal conductivity detector chromatograph and an infrared continuous analyzer. Results show that H_2_ and N_2_ are the main products of the thermal decomposition of ammonia. Under the conditions of the present work, differences between working in a quartz or non-porous alumina reactor are not significant under pyrolysis conditions for temperatures lower than 1400 K. Neither the bath gas nor the ammonia inlet concentration influence the ammonia conversion values. For a given temperature and under all conditions studied, conversion of ammonia increases with an increasing gas residence time, which results into a narrower temperature window for NH_3_ conversion.

## 1 Introduction

Climate change, security of the energy supply, and fossil fuel depletion are well-known issues that determine the need of finding pathways for a transition to a low-carbon economy. The development of technologies for alternative energy sources is a fundamental concern for the transition to a new energetic scenario. In this field, the reduction of the greenhouse gas (GHG) emissions is one of the most investigated issues in the last few years. By 2050, the European Union (EU) aims to reduce its GHG emissions by 80–95% compared to the 1990 levels.^1^ This reduction should be met while also increasing competitiveness and security of the energy supply.

In this context, hydrogen points out as a possible energy carrier. The main advantage of its use is that only water is formed as product in its combustion process. However, hydrogen presents difficulties to be stored and delivered at high pressures. In this scenario, ammonia, which includes hydrogen in its composition, shows up as a viable substitute of non-renewable fuels and as a hydrogen transport vector. Furthermore, ammonia can be mixed with H_2_ or hydrocarbons to increase versatility of its use.^2^

In the last few decades, ammonia has been used as a raw material in the production of fertilizers, nitric acid, plastics, and rubber. This implies that ammonia storage and delivery systems are well-known and affordable. Nowadays, the largest amount of ammonia is produced via the Haber–Bosch process using non-renewable carbon-based raw materials. Nevertheless, the use of a renewable energy, such as solar energy or wind power, to provide the hydrogen requirements in the Haber–Bosch process seems to be technically and economically feasible.^1^ In addition, working under optimum conditions, the ammonia oxidation process exhibits the advantage of generating mainly N_2_ and H_2_O as products. However, apart from the possible NO_*x*_ emissions, the use of ammonia as a fuel presents several disadvantages, related mainly to the oxidation of NH_3_, such as low combustion intensity, low calorific value, and difficulties to ignite.^3^

Several studies have been conducted on the use of mixtures including ammonia and kerosene or other compounds (e.g., CH_4_ and H_2_) as fuel in 10–50 kW burners and turbines with some promising results.^2,4−8^ Likewise, studies of conversion of ammonia and its mixtures, under conditions of interest for combustion, have been performed^9−17^ to increase the knowledge on NH_3_ conversion, to reach the optimum process conditions, and, therefore, to promote the use of ammonia as fuel.

Ammonia decomposition reactions may play a determinant role during ammonia and ammonia mixture oxidation.^3,11−17^ For deeper knowledge about the involved mechanisms, different investigations on ammonia pyrolysis and oxidation in the gas phase have been carried out since 1970 in different kinds of reactors (tubular flow, shock tube, jet stirred, etc.).^3,9−17^ Results indicate that the complex interactions occurring in the NH_*i*_ species chemistry and ammonia decomposition appear to have a dominant role when describing the conversion of NH_3_ under different combustion conditions.^12^ Studies carried out in shock-tube and tubular-flow reactors, at high temperatures, have shown the relevance of reactions R1 and R2(18−23) in thermal decomposition of NH_3_.
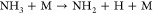
R1
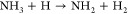
R2Additionally, N_2_H_2_, N_2_H_3_, and N_2_H_4_ species, as intermediates in the ammonia thermal decomposition, have been pointed out to have an important role in kinetic mechanisms describing NH_3_ conversion.^21,22,24^

Despite the importance of the knowledge of ammonia thermal decomposition, few works in this field are presented in the literature. In addition, an aspect of potential relevance that has been addressed in a number of works is the possible impact of the reactor material, which could be determinant in the ammonia pyrolysis reactions at low temperatures.^25^ Some investigations about the surface effect and the adsorption of ammonia and the amidogen radical (NH_2_) on oxide surfaces have been carried out.^25−29^ The amidogen radical (NH_2_) was found to adsorb on quartz and stainless-steel reactors.^26^ Those results may indicate that the reactor material effect could be important in the oxidation of ammonia at low temperatures. Moreover, the results obtained by Manna et al.^29^ from ammonia pyrolysis and oxidation experiments in a ceramic reactor and a quartz flow reactor were significantly different for the different reactor materials. Nevertheless, there is no consensus about the influence of the reactor material on the ammonia decomposition. For this reason, a deeper investigation of the influence of the material surface of the reactor on the decomposition of ammonia is desired.

Given the importance of the ammonia pyrolysis process, it has been considered interesting to present experimental results of the influence of different variables on the ammonia conversion in the absence of any oxidizing agent. This can complement the few experimental results presented in the literature on this topic and contribute to a better knowledge of the process.

In this context, the aim of the present work is to experimentally study at different temperatures the influence of the reactor material (quartz or non-porous alumina), the bulk gas (N_2_ or Ar), the ammonia inlet concentration (1000 and 10 000 ppm), and the gas residence time [2060/*T* (K)–8239/*T* (K) s] on the conversion of ammonia in tubular-flow reactors.

## 2 Experimental Section

**Figure 1:**
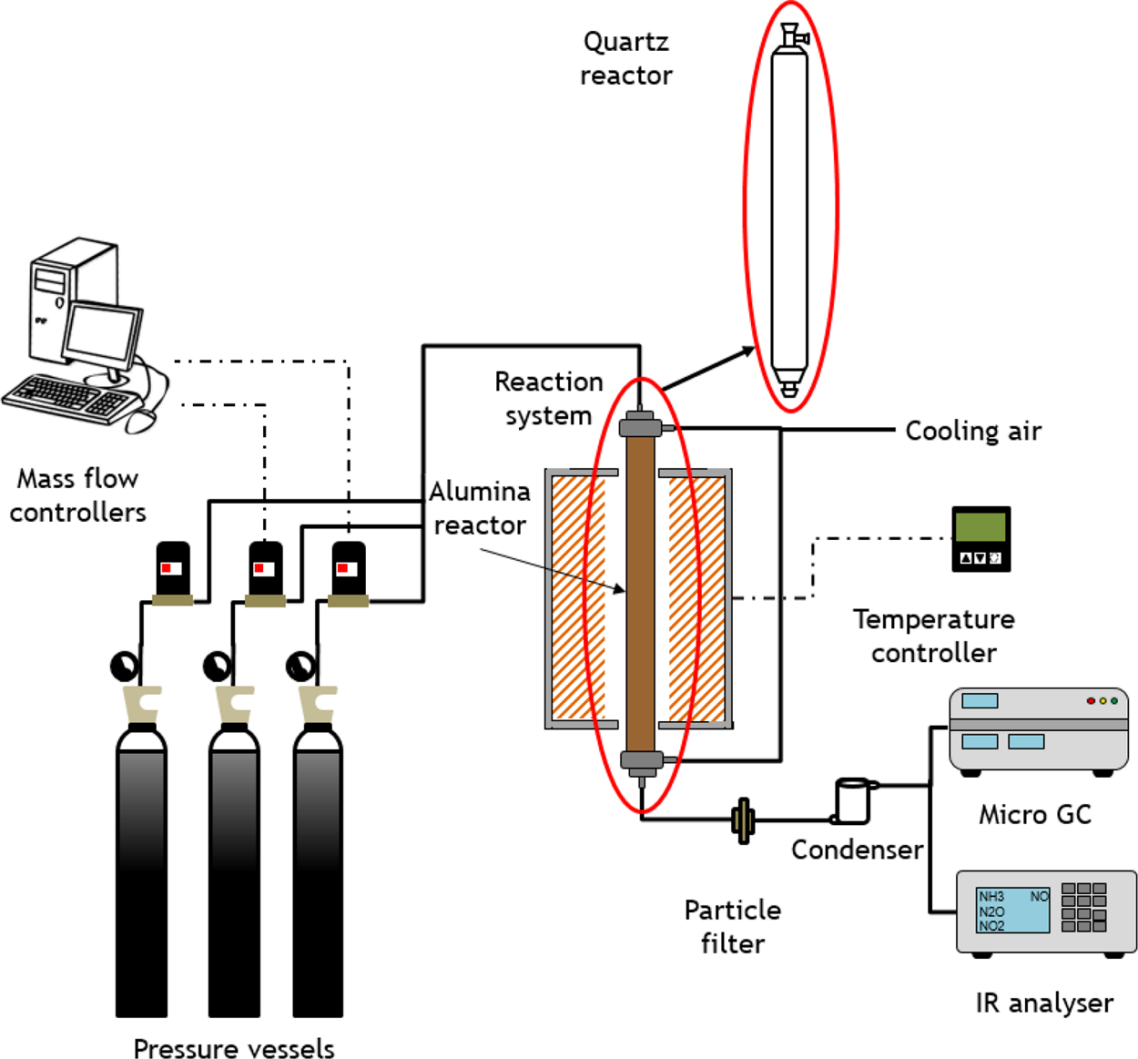
Scheme of the experimental setup, with a quartz or alumina reactor, used for studying the conversion of ammonia under pyrolysis conditions.

The experiments using NH_3_ diluted in argon or nitrogen (<1% NH_3_) have been carried out in an experimental installation (Figure 1), which has been described in detail elsewhere (e.g., refs (30−32)). It consists basically of a gas feeding system, a reaction system working at atmospheric pressure, and a gas analysis system. Gases are supplied from gas cylinders through mass flow controllers, and all reactants are premixed just prior to the reactor inlet. The mass flow controllers present a full-scale error of 1.5%. However, each flow rate was manually checked with a flow meter, reducing the uncertainty of the flows to negligible values.

**Figure 2:**
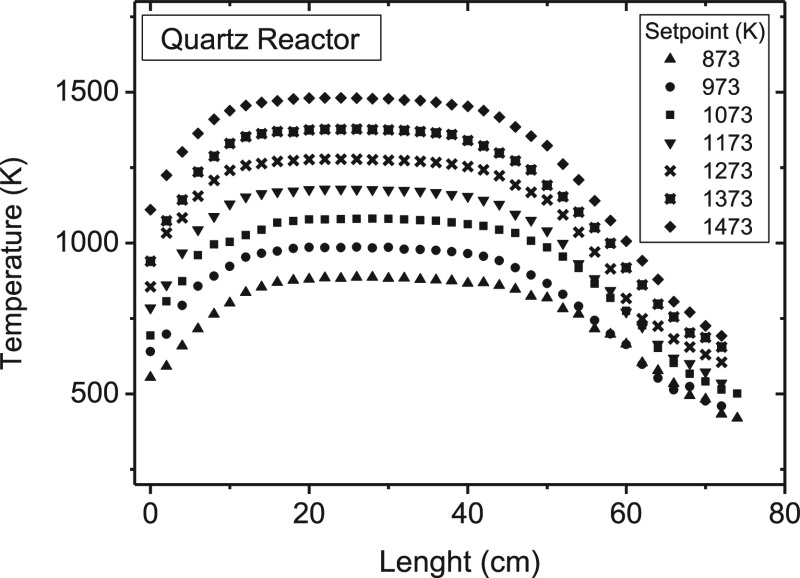
Measured temperature profiles along the quartz reactor in the 873–1473 K range, with the total gas flow rate of 1.0 L of N_2_ (STP)/min.

As mentioned, the installation is equipped with two similar reactors, whose difference is their building material: non-porous alumina (DEGUSSIT AL23, open porosity of 0) or quartz (fused silica, POBEL), which implies the possibility of using two different temperature intervals: 900–1800 K for the non-porous alumina reactor and 900–1500 K for the quartz reactor. Both reactors have the same internal diameter (40 mm) and length (1000 mm) and have previously been used with success in other studies carried out by the research group with other fuels (e.g., refs (30−32)). The longitudinal temperature profiles in the reactor were measured at non-reacting conditions with a type-S ceramic-covered platinum thermocouple placed inside the reactor, for the different total gas flow rates used in this work [0.5, 1.0, 1.5, and 2.0 L standard temperature and pressure (STP)/min] and using both bulk gases (N_2_ and Ar). As an example, the longitudinal measured temperature profiles for the quartz reactor using a flow rate of 1.0 L (STP)/min of N_2_ are shown in Figure 2. Similar temperature profiles were obtained for the other flow rates (section S1 and Figures S1–S3 of the Supporting Information). It is observed that an almost isothermal temperature (within ±10 K) is attained throughout the main reaction zone (200 mm in length, approximately). The isothermal reaction zone obtained for the alumina reactor also had the same length. The profiles determined with a N_2_ flow are very similar to those obtained for Ar, with temperature differences lower than 2 K for a given set point temperature. In all of the experiments, ammonia is highly diluted; thus, the thermal effects during the pyrolysis are small, and the temperature profiles measured, in the absence and presence of the reaction, can be assumed to be the same.

At the outlet of the reactor, the product gas is cooled by means of an external refrigeration with compressed air and analyzed by different analyzers. The analysis equipment used for the experiments include a micro gas chromatograph (micro GC) with a thermal conductivity detector (TCD) to determine N_2_ and H_2_ and an infrared (IR) continuous analyzer to quantify the NH_3_ concentration. The uncertainty of the measurements is estimated as ±5%. No oxygen compounds were detected.

Table 1 shows the conditions of experiments performed, including the gas residence time in the isothermal zone for the different flow rates. For each set, results at different temperatures (increasing the temperature by 50 K in the temperature range shown in Table 1) are obtained.

**Table 1:** Experimental Conditions

set	reactor material	temperature range (K)	gas flow rate [L (STP)/min]	gas residence time (s)	ammonia concentration (ppm)	bulk gas
1	quartz	900–1475	0.5	8239/*T* (K)	992	Ar
2	alumina	900–1800	0.5	8239/*T* (K)	1015	Ar
3	quartz	900–1475	1.0	4119/*T* (K)	1033	Ar
4	alumina	900–1800	1.0	4119/*T* (K)	994	Ar
5	quartz	900–1475	1.5	2746/*T* (K)	993	Ar
6	alumina	900–1800	1.5	2746/*T* (K)	985	Ar
7	quartz	900–1475	2.0	2060/*T* (K)	994	Ar
8	alumina	900–1800	2.0	2060/*T* (K)	1019	Ar
9	quartz	900–1475	1.0	4119/*T* (K)	984	N_2_
9R	quartz	900–1475	1.0	4119/*T* (K)	998	N_2_
10	alumina	900–1800	1.0	4119/*T* (K)	1025	N_2_
11	quartz	900–1475	1.0	4119/*T* (K)	10005	Ar

For the conditions of set 9 in Table 1 and temperatures over the NH_3_ conversion temperature range (results shown in Figure 4), the experiments have been carried out in duplicate, set 9R. The results are also shown in Figure 4. In this way, two results were obtained for each temperature. Assuming that the experimental error does not depend upon the temperature in the interval considered, the pooled standard deviation (the square root of the sum of squares of the error) has been calculated as an estimator of the experimental error associated with the conversion of ammonia (%) in the study area. The pooled standard deviation obtained is 0.4%. Because this pooled standard deviation was calculated considering the ammonia conversion data in percent, the units of this pooled standard deviation are in percent. This pooled standard deviation enables the calculation of the error bars as the 95% confidence intervals for the mean value associated with each repeated experiment, for the average ammonia conversion values of sets 9 and 9R, in Figure 4.

A nominal concentration of 1000 ppm of NH_3_ is introduced to the reaction system, except for set 11 examining the influence of the inlet ammonia concentration, where the nominal concentration corresponds to 10 000 ppm (set 11 in Table 1). A gas flow rate of 1.0 L (STP)/min is also usually used (sets 3, 4, and 9–11 in Table 1), with the exception of the experiments used for the gas residence time influence study, where is varied from 0.5 to 2.0 L (STP)/min. For each set, experiments are performed at different temperatures, and, therefore, for a given gas flow rate, the gas residence time depends upon the temperature, as shown in eq 1.
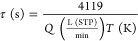
1Table 1 shows this dependence corresponding to the different flow rates. The bulk gas used for most of the experiments is argon (purity of 99.999, quality Alphagaz 1, distributor Air Liquide), except for those experiments where the bulk gas influence is studied (sets 9–10 in Table 1), where N_2_ (purity of 99.999, quality Alphagaz 1, distributor Air Liquide) is used as bulk gas. Argon is mainly used in order to determine N_2_ produced in the pyrolysis reaction. In the experiments performed with N_2_ as bulk gas and given the ammonia-diluted conditions, nitrogen formed is negligible compared to total N_2_.

In all of the experiments with Ar as bulk gas, the H and N atom balance closes between 95 and 108%. For the experiments where N_2_ was used as a bath gas, the H atom balance also ranges between 95 and 106%.

## 3 Results

As was previously mentioned, more research of ammonia thermal decomposition is needed to contribute to explain the ammonia combustion behavior. For this reason, the aim of the experiments performed is to study the influence of different operating variables on the conversion of ammonia under pyrolysis conditions. Under the studied conditions, general results show that ammonia pyrolysis at atmospheric pressure begins at 1173 K. N_2_ and H_2_ are detected as products when ammonia starts to decompose, and the complete decomposition of ammonia can only be observed in the alumina reactor that tolerates higher temperatures compared to the quartz reactor.

Although the objective of the present work is the experimental study of the influence of different variables on the ammonia conversion in its thermal decomposition, it has also been considered interesting to compare some experimental results to those obtained with a mechanism proposed in the literature. The mechanism chosen is that proposed by Song et al.^12^ Simulations were performed using the plug-flow reactor (PFR) code of Chemkin-Pro^33^ and the temperature profiles existing in the reactor. The results obtained, with their corresponding discussion, are presented in section S2 and Figures S4–S6 of the Supporting Information.

### 3.1 Influence of the Reactor Material

**Figure 3:**
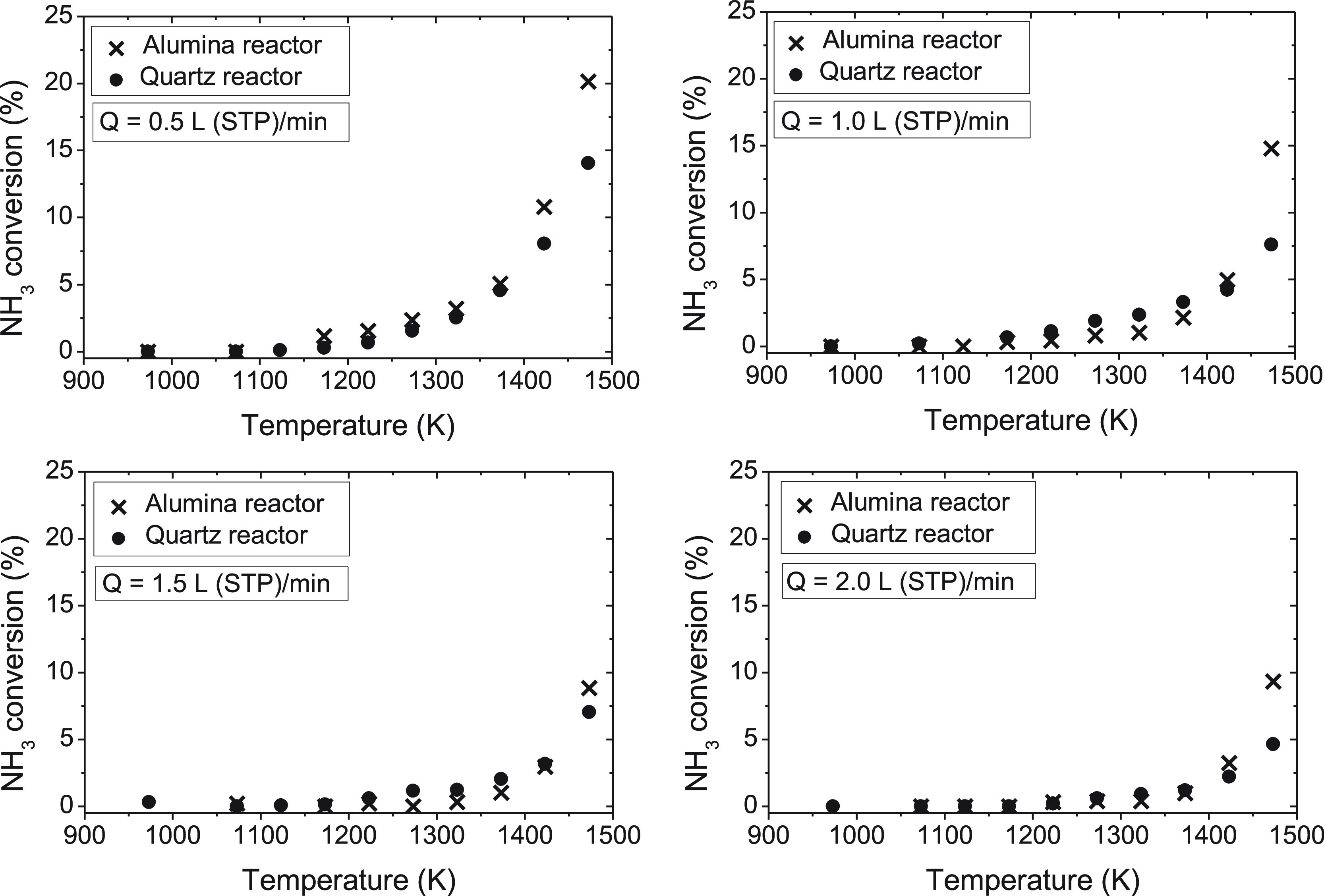
Comparison of ammonia conversion (%) in quartz and non-porous alumina tubular-flow reactors, in the range of 900–1500 K, for different gas flow rates [from 0.5 to 2.0 L (STP)/min] and using argon as bulk gas (sets 1–8 in Table 1).

The influence of the reactor material has been analyzed by a comparison between the results obtained in both reactors (quartz and non-porous alumina), for the different gas flow rates. Figure 3 shows the results of NH_3_ conversion, as a function of the temperature, for gas flow rates of 0.5, 1.0, 1.5, and 2.0 L (STP)/min.

The ammonia conversion obtained seems to be the same in both the quartz and alumina reactors for temperatures *T* ≤ 1400 K, while at 1473 K, conversion of NH_3_ appears to be higher for the experiments carried out in the alumina reactor.

Because, in general, experimental results of NH_3_ conversion obtained under pyrolysis conditions are very similar independently of the reactor material used (quartz or non-porous alumina) for temperatures below 1400 K, this seems to indicate that surface reactions are not important with the reactor materials used and under the mentioned conditions.

One would expect an increased effect of the reactor surface at low temperatures where the extent of reaction is lower. Because, only at the highest temperature, a difference of the results is found, we can rule out the existence of the surface effect at temperatures lower than 1400 K. This does not mean that, at 1473 K and above, surface effects are important.

Glarborg et al.^24^ suggested that surface decomposition could play a determinant role on the systems that react slowly, such as NH_3_ oxidation. Surface effects were observed in the thermal deNO_*x*_ process by Lyon and Benn^34^ but have not been reported in a large amount of the literature. Both ammonia oxidation and the thermal deNO_*x*_ process involve nitrogen and oxygen species in their reaction process. However, in the pyrolysis of ammonia, no oxygen compounds are present; thus, results presented in this work just involved hydrogen and nitrogen species. Figure 3 indicates that differences in the conversion of ammonia under pyrolysis conditions, for the two different reactor materials used in this work, are not significant for lower temperatures than 1400 K.

For the study of the surface influence on the ammonia pyrolysis, Manna et al.^29^ performed pyrolysis experiments in two laminar flow reactors of different building materials (quartz and alumina). For temperatures higher than 1150 K, the hydrogen and nitrogen concentration values changed significantly for the different reactor materials tested, an effect that has not been observed in the present work.

### 3.2 Influence of the Bulk Gas

A study of the bulk gas effect on the thermal decomposition of ammonia has been performed, using N_2_ or Ar as bulk gas in both reactors, the quartz reactor and the non-porous alumina reactor, and with a total gas flow rate of 1.0 L (STP)/min and a nominal NH_3_ concentration of 1000 ppm (sets 3, 4, and 9–10 in Table 1).

**Figure 4:**
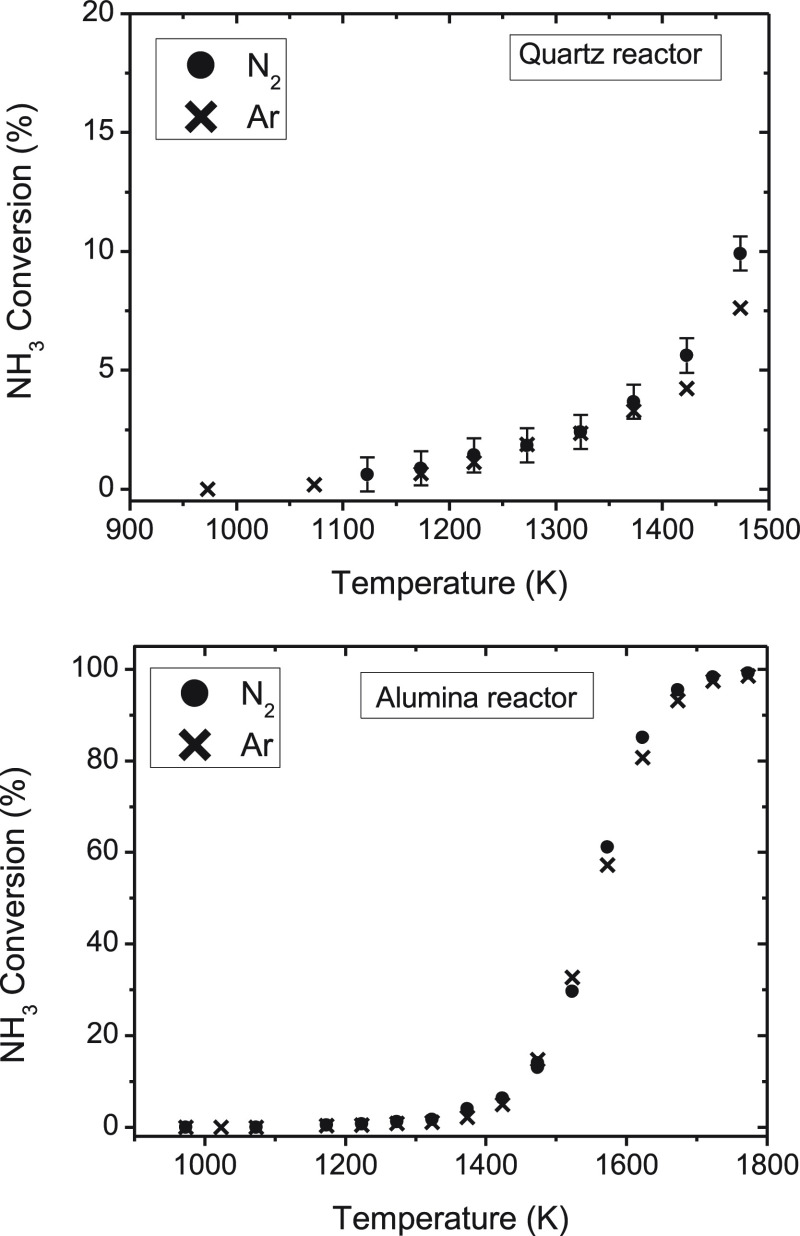
Influence of the bulk gas, Ar (sets 3 and 4 in Table 1) or N_2_ (sets 9, 9R, and 10 in Table 1) on the ammonia conversion (%) in a quartz tubular-flow reactor (900–1500 K) and in an alumina tubular-flow reactor (900–1800 K), with the total gas flow rate of 1.0 L (STP)/min.

**Figure 5:**
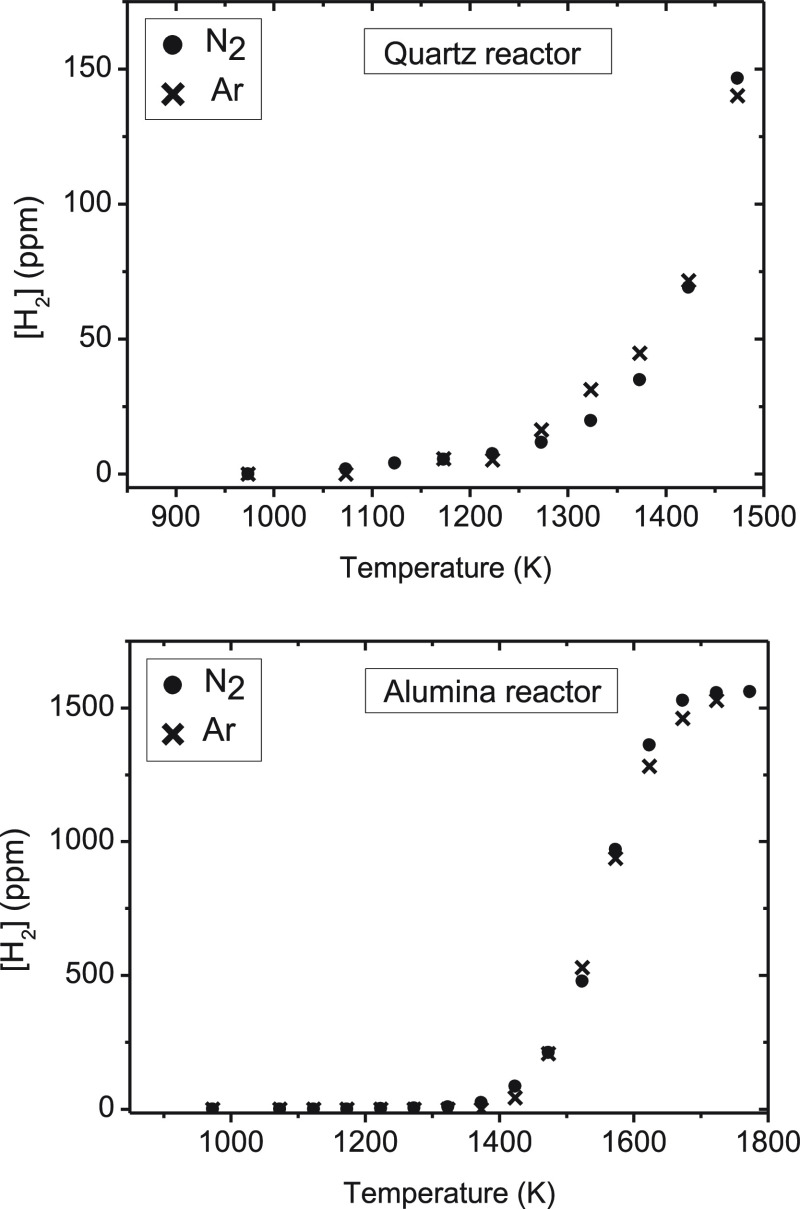
Influence of the bulk gas, Ar (sets 3 and 4 in Table 1) or N_2_ (sets 9 and 9R in Table 1) on H_2_ formed (ppm) in a quartz tubular-flow reactor (900–1500 K) and in a non-porous alumina tubular-flow reactor (900–1800 K), with a total flow rate of 1.0 L (STP)/min.

The results obtained in these experiments are shown in Figures 4 and 5. Figure 4 includes the results of NH_3_ conversion (%), and Figure 5 includes the results of H_2_ formed (ppm), in the quartz and alumina reactors, separately. There are not significant differences between using N_2_ or Ar as the bath gas for ammonia pyrolysis in any of reactors. The quartz reactor results (Figure 4) show the highest conversion of NH_3_ at 1473 K. No experiments at higher temperatures were performed in the quartz reactor as a result of the vitreous transition temperature of quartz around 1500 K. The results obtained in the alumina reactor (Figure 4) show the same trend using Ar or N_2_ as bulk gas, reaching the complete decomposition of ammonia at 1700 K. In the experiments carried out in the presence of argon, N_2_ formed was quantified and the nitrogen atomic balance closed within 95–106% (mentioned above), indicating the good performance of the experiments.

The results of the concentration of hydrogen formed in the pyrolysis of ammonia in the quartz reactor and in the alumina reactor, using N_2_ and Ar as bath gas, are presented in Figure 5. No appreciable effect of the bath gas is found on the hydrogen concentration values.

### 3.3 Influence of the NH_3_ Inlet Concentration

**Figure 6:**
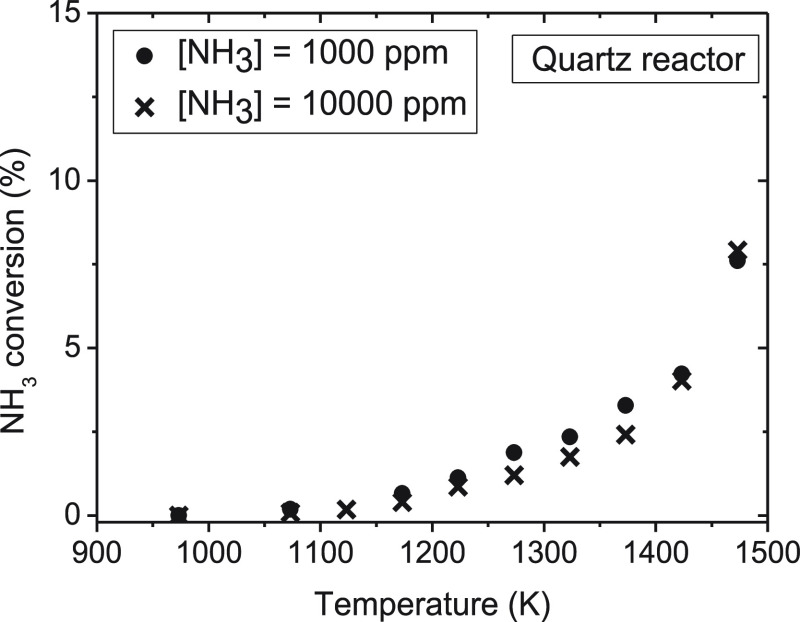
Influence of the NH_3_ inlet concentration on the ammonia conversion (%) in a quartz tubular-flow reactor, in the range of 900–1500 K, for two NH_3_ concentrations (sets 3 and 11 in Table 1), with a total gas flow rate of 1.0 L (STP)/min.

The influence of the inlet concentration of ammonia on the products obtained during NH_3_ thermal decomposition has been studied in the quartz reactor, using two different ammonia nominal concentrations, 1000 and 10 000 ppm, and a total gas flow rate of 1.0 L (STP)/min (sets 3 and 11 in Table 1). To determine the N atom balance, Ar was used as bulk gas in these experiments. The comparison of conversion of ammonia for the two inlet ammonia concentrations studied is shown in Figure 6. No significant effect of the inlet concentration of ammonia is found on the conversion of this species, with slightly higher conversions (with a maximum difference of 0.7%) observed for the lowest ammonia concentration considered.

**Figure 7:**
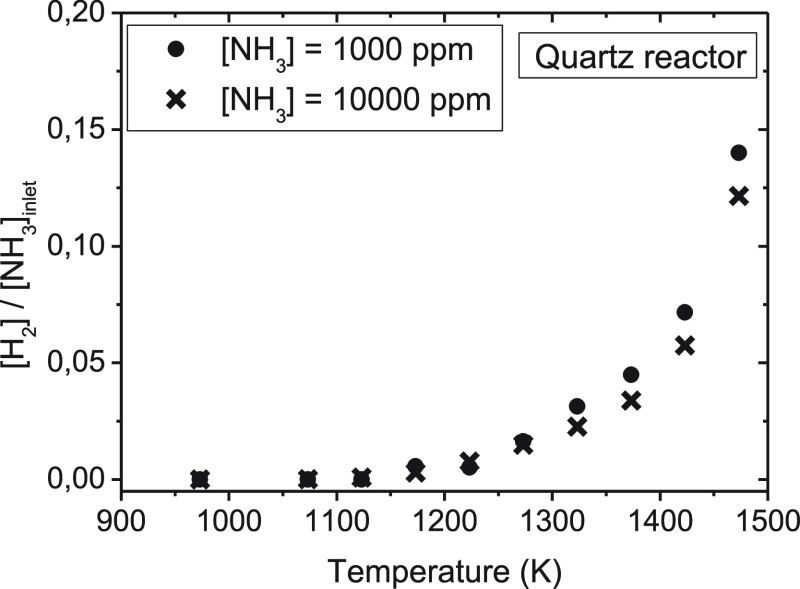
Hydrogen formed (ppm) in a quartz tubular-flow reactor, in the range of 900–1500 K, for inlet concentrations of ammonia of 1000 ppm (set 3 in Table 1) and 10 000 ppm (set 11 in Table 1), with a total gas flow rate of 1.0 L (STP)/min.

The concentration of H_2_ formed in the experiments is shown, as the normalized concentration, i.e., [H_2_]_outlet_/[NH_3_]_inlet_, in Figure 7. Between 1250 and 1450 K, slight differences (up to 0.02 as the maximum) in the [H_2_]_outlet_/[NH_3_]_inlet_ ratio can be appreciated. The effect of the inlet ammonia concentration appears not to be very significant on the results obtained.

### 3.4 Influence of the Gas Residence Time

**Figure 8:**
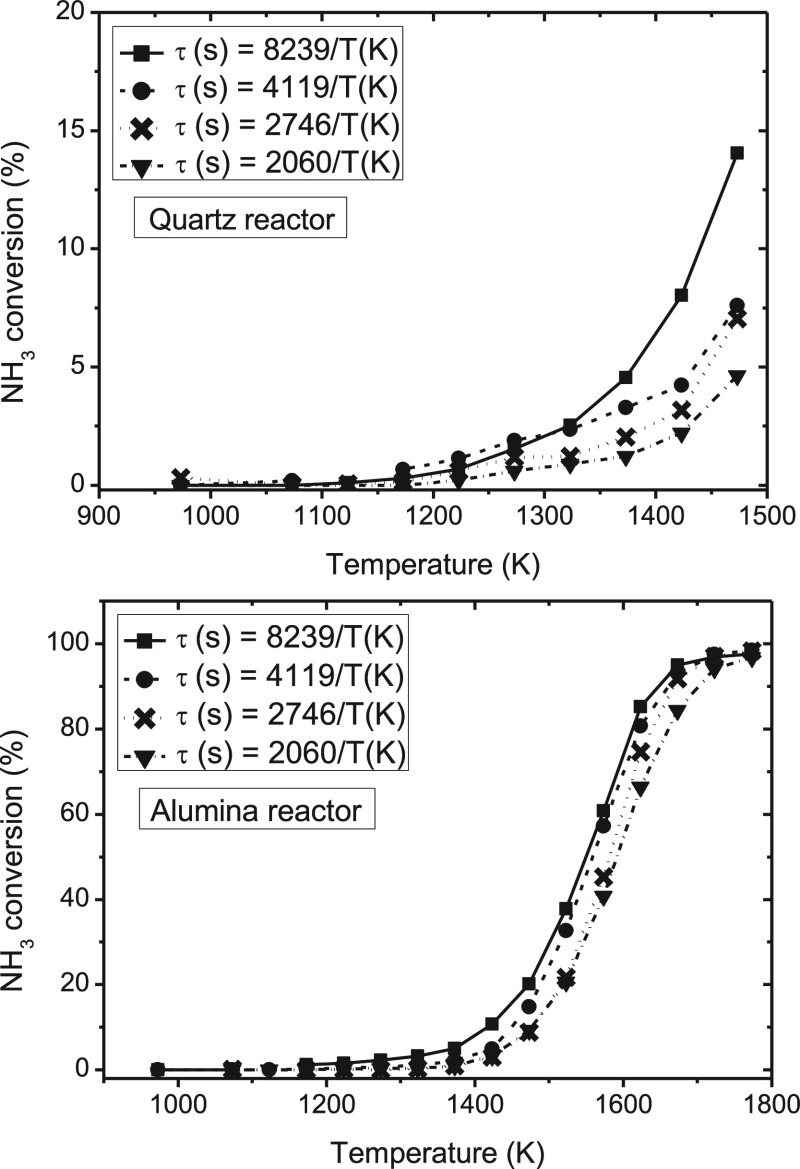
Influence of the gas residence time on the ammonia conversion (%) in a quartz tubular-flow reactor (900–1500 K) and in an alumina tubular-flow reactor (900–1800 K; sets 1–8 in Table 1).

**Figure 9:**
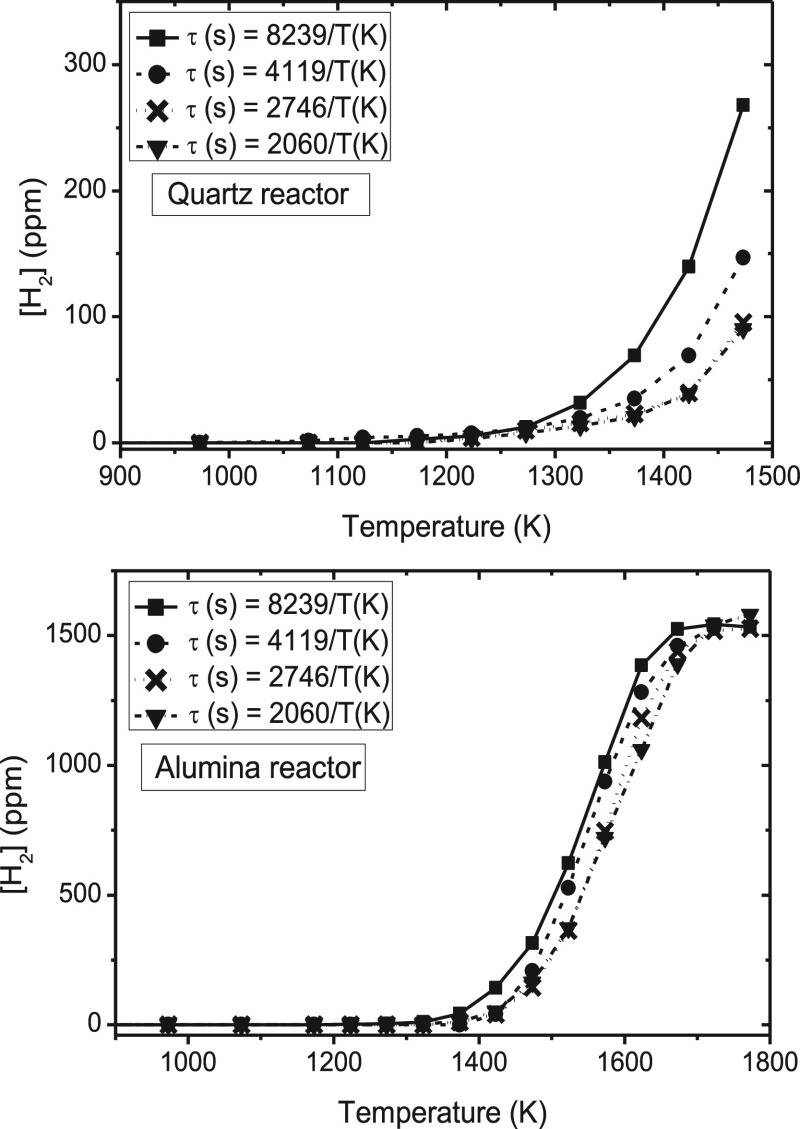
Influence of the gas residence time on the concentration of H_2_ formed (ppm) in a quartz tubular-flow reactor (900–1500 K) and in an alumina tubular-flow reactor (900–1800 K; sets 1–8 in Table 1).

For the evaluation of the influence of the gas residence time on the conversion of ammonia under pyrolysis conditions, four different sets of experiments have been carried out in each reactor (sets 1–8 in Table 1). The experiments mainly consist of varying the inlet gas flow rate in the experiments while keeping the nominal inlet concentration of NH_3_ constant at 1000 ppm. The results of NH_3_ conversion and H_2_ concentration obtained are shown in Figures 8 and 9, respectively. The experimental points have been joined with lines to facilitate the analysis. It should be noted, as mentioned in the Experimental Section, that for a given gas flow rate, in each experiment, the temperature is varied in the corresponding temperature interval considered, thus resulting in a residence time dependent upon the temperature (eq 1).

As previously indicated, as a result of temperature limitations, the results of ammonia conversion in the quartz reactor only cover up to 1500 K, where low NH_3_ conversions are obtained. In the alumina reactor, full conversion of ammonia is attained at the highest temperatures analyzed.

The conversion of ammonia increases with the gas residence time (by decreasing the flow rate) in both reactors. However, the gas residence time does not modify the initiation temperature for NH_3_ conversion, which is attained at around 1200 K. For lower temperatures than 1700 K, the conversion of NH_3_ increases with an increase in the gas residence time.

The influence of the gas residence time on the conversion of ammonia under pyrolysis conditions in a quartz flow reactor was studied previously by Monnery et al.^35^ In their study, the variation of the residence time was achieved using different reactor lengths. Results showed a relation between the conversion of ammonia and the gas residence time. An increase in the conversion of ammonia was related to an increase in the gas residence time (keeping the temperature constant). Experiments carried out in this work also show an increase in the conversion for the higher gas residence time tested.

## 4 Conclusion

A study of ammonia pyrolysis at atmospheric pressure, at different temperatures, in two reactors of different material (quartz and non-porous alumina), at different gas residence times, and using either Ar or N_2_ as bath gas, has been performed. For all of the experiments, a diluted ammonia concentration was used. The quartz reactor experiments were performed in the temperature range of 900–1500 K, while the temperature range was extended in the alumina reactor, where it was possible to operate up to 1800 K.

Results show that, independent of the operating conditions and atmosphere considered in the present work, the pyrolysis of ammonia begins at approximately 1173 K. In all of the experiments with Ar as bulk gas, the H and N atom balance closes between 95 and 108%. For the experiments where N_2_ was used as a bath gas, the H atom balance also ranges between 95 and 106%.

A study of the influence of reactor material on NH_3_ pyrolysis has been carried out comparing the thermal decomposition of ammonia in quartz and non-porous alumina reactors. Conversion of ammonia in both reactors shows similar values and trends for lower temperatures than 1400 K, which points to no significant influence of surface effects under the mentioned conditions. An appreciable difference can be seen for the highest temperatures tested in the quartz reactor (around 1500 K).

Under the conditions of the present work, no significant influence of the bath gas can be appreciated on the conversion of ammonia operating in either the quartz reactor or alumina. Two different inlet concentrations of ammonia were tested using a flow rate of 1.0 L (STP)/min. Not significant differences in the ammonia conversion were observed using inlet concentrations of ammonia of 1000 and 10 000 ppm.

To study the influence of the gas residence time, four different gas flow rates were tested from 0.5 to 2.0 L (STP)/min. For the different gas residence time studied, the pyrolysis of ammonia begins around 1200 K. For a given temperature, conversion of ammonia increases with an increase of the gas residence time, under all conditions studied, which results into a narrower temperature window for NH_3_ conversion. The full conversion of ammonia in the alumina reactor is obtained at 1700 K, independent of the gas residence time considered.

The present results are valuable for assessing the different issues affecting the studies dealing with ammonia conversion under laboratory conditions.

## Supporting Information Available

Longitudinal temperature profiles in the quartz reactor (section S1), measured temperature profiles along the quartz reactor in the 973–1473 K range, with a total flow rate of 0.5 L of N_2_ (STP)/min (Figure S1), measured temperature profiles along the quartz reactor in the 973–1473 K range, with a total flow rate of 1.5 L of N_2_ (STP)/min (Figure S2), measured temperature profiles along the quartz reactor in the 873–1373 K range, with a total flow rate of 2.0 L of N_2_ (STP)/min (Figure S3), simulation of the experimental results (section S2), experimental and simulated ammonia conversion in an alumina tubular-flow reactor (900–1800 K; set 4 in Table 1) (Figure S4), NH_3_ sensitivity analysis for a temperature of 1400 K (Figure S5), and NH_3_ pyrolysis reaction pathways for a temperature of 1400 K (Figure S6) (PDF)
