# Supplementary material for: Experimental Study of the Pyrolysis of NH3 under Flow Reactor Conditions
Source: Energy Fuels. 2021 Jan 20;35(9):7193–200. doi: 10.1021/acs.energyfuels.0c03387 (PMC9165062; doi:10.1021/acs.energyfuels.0c03387)
Supplement: Supplementary file 1 — ef0c03387_si_001.pdf [file ef0c03387_si_001.pdf]

Supplementary material

Experimental study of the pyrolysis of NH_3 under flow reactor conditions

*Benés M., Pozo G., Abián M., Millera A., Bilbao R. and Alzueta M.U.**

Aragón Institute of Engineering Research (I3A). Department of Chemical and Environmental Engineering, University of Zaragoza, 50018 Zaragoza, Spain.

Section S1. Longitudinal temperature profiles in the quartz reactor

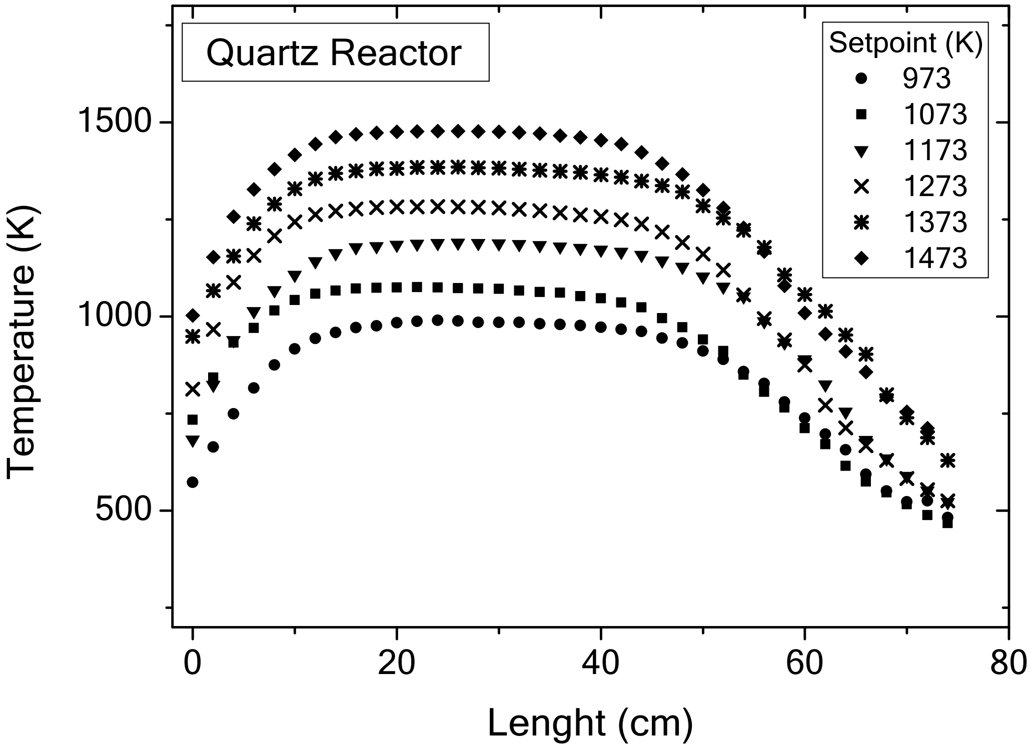

Figure S1. Measured temperature profiles along the quartz reactor in the 973–1473 K range. Total flow rate of 0.5 L N_2 (STP)/min.

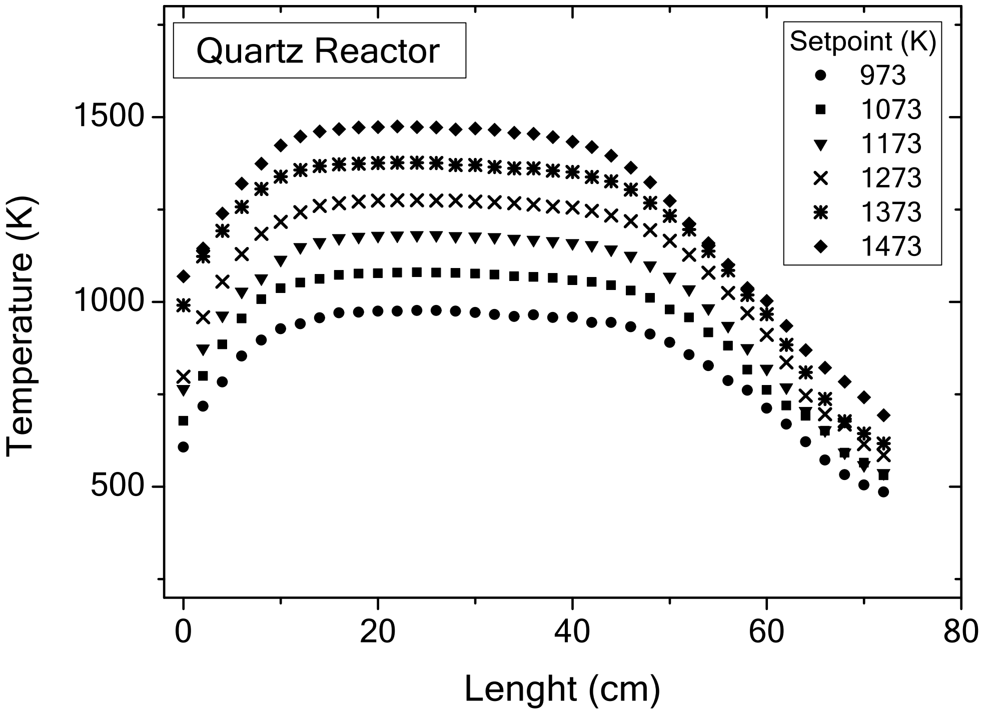

Figure S2. Measured temperature profiles along the quartz reactor in the 973–1473 K range. Total flow rate of 1.5 L N_2 (STP)/min.

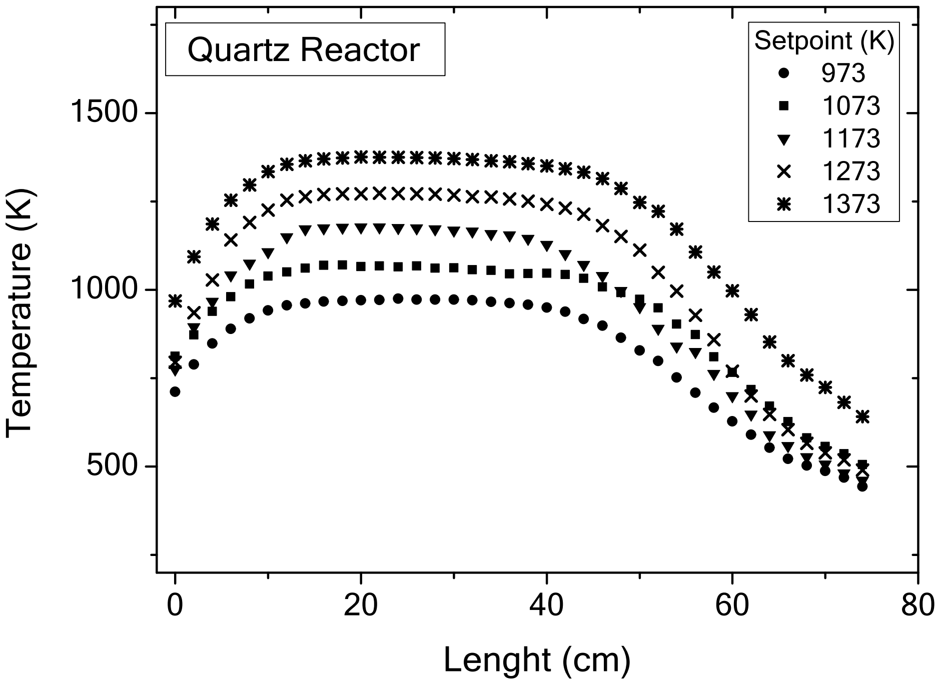

Figure S3. Measured temperature profiles along the quartz reactor in the 873–1373 K range. Total flow rate of 2.0 L N_2 (STP)/min.

Section S2. Simulation of the experimental results

Experimental results obtained in the alumina flow reactor for a flow rate of 1.0 L (STP)/min (set 4 in Table 1) were simulated using the Song et al. mechanism [12]. Simulations were performed using the plug flow reactor (PFR) code of CHEMKIN PRO [33]. Figure S4 shows the results obtained in the simulations. The mechanism proposed by Song et al. [12] predicts well the ammonia conversion for temperatures lower than 1473 K under the conditions studied in this set (set 4 in Table 1). For temperatures higher than 1473 K, the calculations show a lower conversion than that experimentally obtained. The complete decomposition of ammonia is reached at 1773 K.

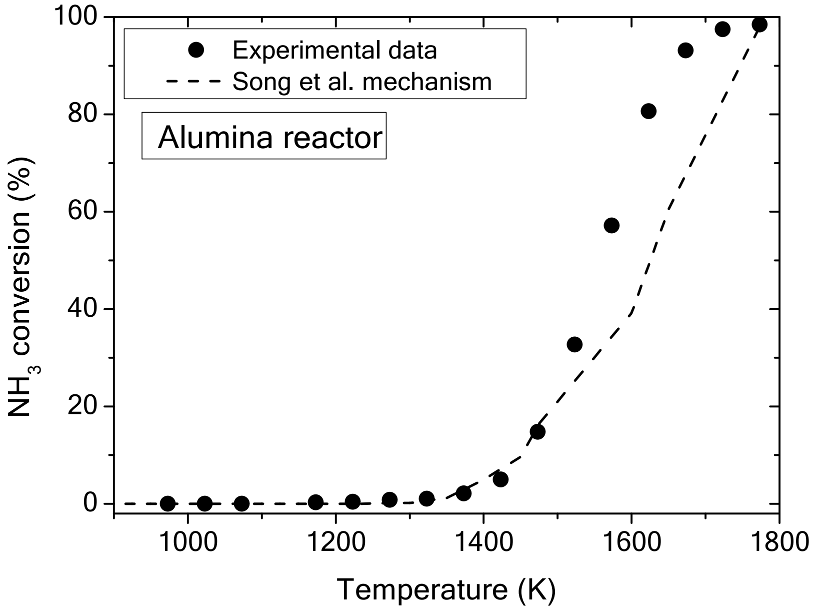

Figure S4. Experimental and simulated ammonia conversion in an alumina tubular-flow reactor (900-1800 K), (set 4 in Table 1).

In addition, Figure S5 shows the sensitivity analysis of the ammonia decomposition reactions at a temperature of 1400 K. According to the model proposed by Song et al. [12], the conversion of ammonia under the studied conditions is mainly influenced by the $NH_3 + M \rightleftarrows NH_2 + H + M$ reaction (r.S1). The values of the modified Arrhenius parameters in the Song et al. mechanism were proposed by Davidson et al. [22].

$$NH_3 + M \rightleftarrows NH_2 + H + M \qquad \text{(r.S1)}$$

Sensitivity analysis shows that apart of the $NH_3 + M \rightleftarrows NH_2 + H + M$ reaction (r.S1), the maximum sensitivity reactions involved on the decomposition of ammonia were the following reactions:

$$N_2H_2 + M \rightleftarrows NNH + H + M \qquad \text{(r.S2),}$$

$$NH_2 + NH \rightleftarrows N_2H_2 + H \qquad \text{(r.S3),}$$

$NH_2 + NH_2 \rightleftarrows N_2H_2 + H_2$ (r.S4),

$NH_2 + NH_2 \rightleftarrows NH_3 + NH$ (r.S5),

$NH_3 + H \rightleftarrows NH_2 + H_2$ (r.S6).

These indicate that N_2H_2 plays an important role on the ammonia pyrolysis decomposition. Otherwise, the maximum sensitivity reactions involved on the formation of ammonia under pyrolysis conditions were:

$N_2H_2 + NH_2 \rightleftarrows NNH + NH_3$ (r.S7),

$N_2H_2 + H \rightleftarrows NNH + H_2$ (r.S8),

$NH_2 + NH \rightleftarrows NH_3 + N$ (r.S9),

$NH + H \rightleftarrows N + H_2$ (r.S10).

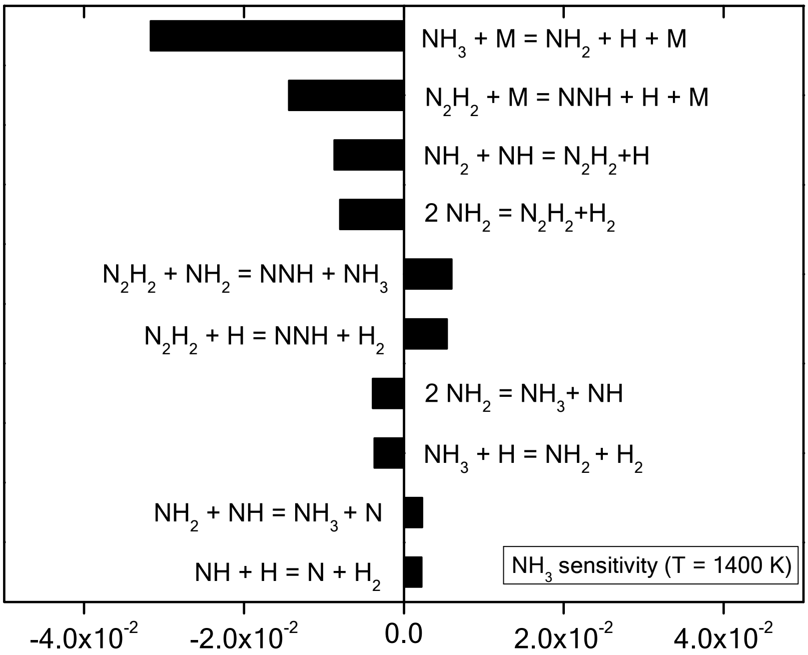

Figure S5. NH_3 sensitivity analysis for a temperature of 1400 K.

Both, the main reaction pathways, shown in Figure S6, and the sensitivity analysis (Figure S5) point out the important role of NH_2, NH, NNH and N_2H_2 species in the ammonia pyrolysis mechanism.

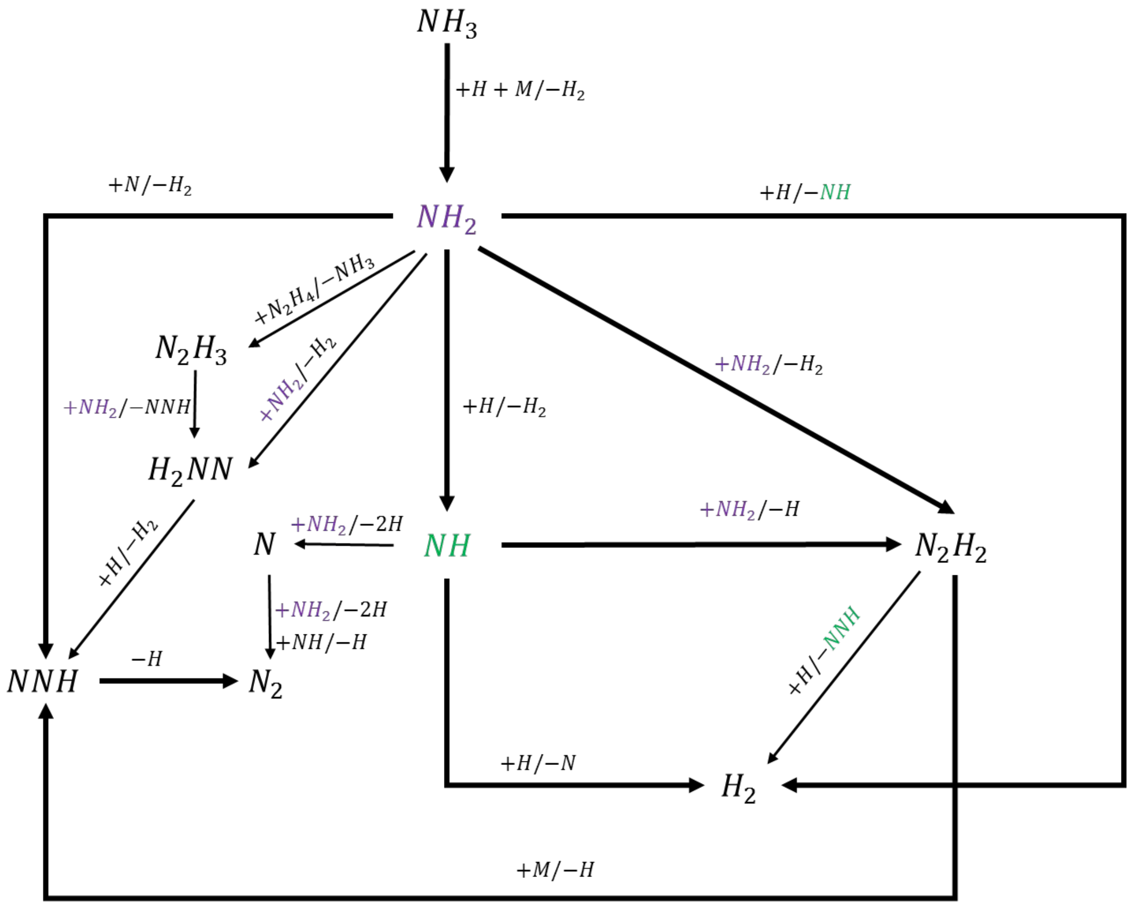

Figure S6. NH_3 pyrolysis reaction pathways for a temperature of 1400 K.
